# Quantification and Dynamic Monitoring of *EGFR* T790M in Plasma Cell-Free DNA by Digital PCR for Prognosis of EGFR-TKI Treatment in Advanced NSCLC

**DOI:** 10.1371/journal.pone.0110780

**Published:** 2014-11-18

**Authors:** Zhijie Wang, Rui Chen, Shuhang Wang, Jia Zhong, Meina Wu, Jun Zhao, Jianchun Duan, Minglei Zhuo, Tongtong An, Yuyan Wang, Hua Bai, Jie Wang

**Affiliations:** Department of Thoracic Medical Oncology, Key Laboratory of Carcinogenesis and Translational Research (Ministry of Education), Beijing Cancer Hospital & Beijing Institute for Cancer Research, Beijing, China; University of Barcelona, Spain

## Abstract

**Background:**

Among advanced non-small cell lung cancer (NSCLC) patients with an acquired resistance to epidermal growth factor receptor-tyrosine kinase inhibitors (EGFR-TKI), about 50% carry the T790M mutation, but this frequency in EGFR-TKI-naïve patients and dynamic change during therapy remains unclear. This study investigated the quantification and dynamic change of T790M mutation in plasma cell-free DNA (cf-DNA) of advanced NSCLC patients to assess the clinical outcomes of EGFR-TKI therapy.

**Materials and Methods:**

We retrospectively investigated 135 patients with advanced NSCLC who obtained progression-free survival (PFS) after EGFR-TKI for >6 months for their EGFR sensitive mutations and T790M mutation in matched pre- and post-TKI plasma samples, using denaturing high-performance liquid chromatography (DHPLC), amplification refractory mutation system (ARMS), and digital-PCR (D-PCR). Real-time PCR was performed to measure c-MET amplification.

**Results:**

Detection limit of D-PCR in assessing the T790M mutation was approximately 0.03%. D-PCR identified higher frequency of T790M than ARMS in pre-TKI (31.3% vs. 5.5%) and post-TKI (43.0% vs. 25.2%) plasma samples. Patients with pre-TKI T790M showed inferior PFS (8.9 vs. 12.1 months, p = 0.007) and overall survival (OS, 19.3 vs. 31.9 months, p = 0.001) compared with those without T790M. In patients harboring EGFR sensitive mutation, high quantities of pre-TKI T790M predicted poorer PFS (p = 0.001) on EGFR-TKI than low ones. Moreover, patients who experienced increased quantity of T790M during EGFR-TKI treatment showed superior PFS and OS compared with those with decreased changes (p = 0.044 and p = 0.015, respectively).

**Conclusion:**

Qualitative and quantitative T790M in plasma cf-DNA by D-PCR provided a non-invasive and sensitive assay to predict EGFR-TKI prognosis.

## Introduction

Inhibition of epidermal growth factor receptor (EGFR) kinase activity by EGFR tyrosine kinase inhibitors (EGFR-TKIs), such as gefitinib and erlotinib, can result in improved response and prolonged progression-free survival (PFS) in selected non-small cell lung cancer (NSCLC) patients harboring sensitizing EGFR mutations, especially the exon 19del and exon 21 L858R mutations [1]–[5]. Unfortunately, almost all patients will ultimately develop resistance to EGFR-TKI, in whom more than 50% cases were detected harboring the EGFR T790M mutation in tumor specimens after EGFR-TKI [6], [7].

T790M mutation was previously regarded as a secondary mutation that was acquired following EGFR-TKI therapy of tumors harboring sensitizing EGFR mutations. Recently, increasing evidences suggested that T790M might co-exist at a low frequency before EGFR-TKI therapy [8], [9]. However, by highly sensitive assays, the frequencies of T790M mutation were reported ranging from 40% to 79% in EGFR-TKI naive NSCLC patients with sensitizing EGFR mutations [10], [11], [12]. The high positive rate of de novo T790M mutation implies an important meaning of exploring the predictive value of pre-TKI T790M mutation status. However, the samples used for T790M detection in previous studies were formalin-fixed paraffin embedded (FFPE) tumor tissue samples, which might confer false positive reported by a recent study [13]. Utilizing fresh/frozen tissue samples for T790M detection is ideal but challenging in clinical practice for advanced NSCLC, especially in dynamic monitoring during therapy. So, exploring supplementary samples and noninvasive assays for T790M detection is needed.

Cell-free DNA (cf-DNA) in plasma is a kind of fresh and real-time sample, and has been shown to be promising for the detection of sensitizing EGFR mutations [14]–[18], which as a noninvasive genotyping method also could facilitate the dynamic monitoring of gene variations including EGFR sensitive and T790M mutations during EGFR-TKI therapy. However, a challenge was also raised about how to detect the low abundance of mutant alleles in plasma cf-DNA. Moreover, it might be important to evaluate T790M quantitatively rather than only qualitatively to optimize personalized therapies. Digital PCR (D-PCR) strategies have been used to accurately estimate the frequency and quantity of sensitizing EGFR mutant alleles [17], [19], which provided a promising and highly sensitive genotyping assays for T790M mutation analysis.

In this study, we used qualitative and quantitative methods, including highly-sensitive D-PCR, to assess the EGFR T790M mutation in plasma DNA samples from patients with advanced NSCLC before and after EGFR-TKI therapy. We then correlated our findings with clinical outcomes.

## Materials and Methods

### Patients and specimens

We retrospectively analyzed 135 advanced NSCLC (stage IIIb or IV) patients who received EGFR-TKI treatment (gefitinib or erlotinib) at the Peking University Cancer Hospital between April 1st, 2005 and July 31st, 2012. Inclusion criteria were: 1) PFS after EGFR-TKI >6 months; and 2) sufficient plasma samples for analyses of EGFR mutations before and after EGFR-TKI treatment. EGFR-sensitive mutations (19del and 21L858R) were analyzed in tumor tissues of 130 patients before EGFR-TKI treatment. We collected the plasma samples when PD after EGFR-TKI was observed but a subsequent treatment did not begin. The interval time between PD after EGFR-TKI and plasma extract was less than 21 days.

PFS after EGFR-TKI was defined as the time interval between beginning EGFR-TKI and disease progression or death. The overall survival (OS) was defined as the time interval between disease diagnosis and death. Clinical data, including age, gender, histological type of cancer, smoking status, imagery and clinical outcomes after EGFR-TKI were reviewed. Light smokers were defined as patients who had smoked less than 100 cigarettes in their lifetime.

The study was approved by the Institutional Review Boards of the Peking University Cancer Hospital. All patients provided written informed consent before the collection and use of their tumor specimens and blood samples.

### EGFR-sensitive mutation detection by denaturing high-performance liquid chromatography and amplification refractory mutation system

Specimen collection, DNA extraction, and denaturing high-performance liquid chromatography (DHPLC) were performed according to methods described in our previous publication [14]. The amplification refractory mutation system (ARMS), as a more sensitive method for sensitizing EGFR mutation, was used to reevaluate the EGFR mutation status identified as EGFR wild-type by DHPLC [1], [20], [21].

### Detection and quantification of T790M mutation by nanofluidic digital PCR array

D-PCR analyses were conducted using the EGFR T790M mutation detection kit (Amoy Diagnostics Company, Haicang, China) and the nanofluidic 12.765 digital array chip (Fluidigm, South San Francisco, CA, USA). Each chip contains 12 individual panels, and each panel contains 765 independent 6-nl reaction chambers. We prepared 10 µl of reaction mixture that contained 8.2 µl of T790M reaction mixture (Amoy), 0.1 µl of Taq polymerase, 0.5 µl of 20×GE sample loading reagent (Fluidigm) and 0.2 µl of 50×6-carboxy-X-rhodamine (ROX) stock solution (Transgene Technology Co., Juegang, China). We then added 1 µl of DNA sample or ddH_2_O, which was used as a negative control. We pipetted 9 µl of the reaction mixture into the chip inlets, and the nanoscale valves and channels in the chip delivered the reaction mixture to the 765 individual reaction chambers. This partitioning of the mixture allowed the target sequences to be amplified separately. We analyzed all matched samples in the same chip in order to standardize the detection conditions. The digital array underwent thermocycling on a BioMark real-time PCR system (Fluidigm). The thermocycling conditions were: 30 s at 50°C, hot start at 95°C for 5 min, and 15 cycles of 95°C for 25 s, 64°C for 20 s, 72°C for 20 s, followed by 31 cycles of 95°C for 25 s, 60°C for 35 s with fluorescence reading (FAM and HEX), and 72°C for 20 s (as suggested by Amoy). The internal reference gene (HEX) is located at the conserved region of exon 2 of EGFR gene.

The D-PCR analysis software was used to process the data and count the numbers of both FAM-positive chambers (mutated T790M) and HEX-positive chambers (the internal reference gene) in each panel. For the T790M mutation assay, a chamber was considered to contain a mutant allele if the mutant signals were positive; a chamber was considered to contain a wild-type sequence if it was positive for the reference signal and negative for the mutated signal. The total numbers of positive chambers for the two signals were then used to estimate the true numbers of mutated T790M alleles in each panel, and to calculate the ratio of mutated T790M/(mutated T790M + wild-type alleles).

### Real-time PCR to detect c-MET amplification

The c-MET copy number was determined by quantitative real-time PCR using a Stratagene Mx3000P Real-Time PCR System (Agilent Technologies, Santa Clara, CA, USA) with TaqMan Universal PCR Master Mix (Applied Biosystems, Foster City, CA, USA). The reference used was TaqMan Copy Number Reference Assay RNase P(4403326), and the MET primer and probe were designed by Applied Biosystems (Hs01432482_cn). Normal human genomic DNA was used as a control (Human TaqMan Control Genomic DNA, 4312660). We defined c-MET gene amplification as: 2^−ΔΔCT^ >1.5 (ΔCT = CT^MET^–CT^RNasp^, ΔΔCT = ΔCT^case^–ΔCT^normal^) [22].

### Statistical analysis

All analyses were performed using SPSS 17.0 (SPSS Inc., Chicago, IL, USA). Comparisons of proportions were performed by χ^2^ tests. The Kaplan-Meier method was used to estimate survival curves for PFS and OS. Log-rank tests were used to compare the survival curves between different subgroups. A two-sided P<0.025 was considered statistically significant.

## Results

### Patients’ characteristics

A total of 135 EGFR-TKI-treated patients with advanced NSCLC were selected for the present study (Table 1), including 50 male and 85 female. Forty-one patients (30.4%) received EGFR-TKI as a first-line treatment, and the others (94/135, 69.6%) underwent EGFR-TKI therapy as second line or beyond. The majority of these patients were never/light smokers (103/135, 76.3%) and with lung adenocarcinoma (130/135, 96.3%). Of these patients, 130 patients have been genotyped for EGFR mutation status in initial tumor tissue specimens: 91 (70%) patients harbored sensitizing EGFR mutation and 39 (30%) carried the wild-type EGFR gene. Among 103 patients with available matching plasma samples before and after EGFR-TKI treatment, 83 cases carried sensitizing EGFR mutation.

**Table 1:** Patients’ characteristics (n = 135).

Characteristics	Patients, n (%)
Age, years	
Median	61
Range	34–79
Gender	
Male	50 (37.0)
Female	85 (63.0)
Smoking status	
Never/light*	103 (76.3)
Former/current	32 (23.7)
Histology	
Adenocarcinoma	130 (96.3)
Non-adenocarcinoma	5 (3.7)
EGFR mutation**	
Mutant type	91 (70.0)
Wild type	39 (30.0)
EGFR-TKI treatment line	
First line	41 (30.4)
Second or beyond	94 (69.6)

*Light smokers referred to patients who had smoked less than 100 cigarettes in their lifetime.

**There were 130 patients who had sufficient tissue for analyzing sensitizing EGFR mutations.

EGFR: epithelial growth factor receptor; TKI: tyrosine kinase inhibitor.

### Determination of detection limit of D-PCR for T790M mutation

To assess the detection limit of D-PCR in detecting T790M mutation, a pilot test was performed using a plasmid carrying T790M or genomic DNA from normal human peripheral-blood mononuclear cells (PBMCs). When the probe specific for the T790M mutation or the human genomic DNA was used separately, the dots were distinctly visualized as red (the FAM fluorescence signal for T790M mutation) or blue (the HEX signal for control), but not vice versa.

**Figure 1:**
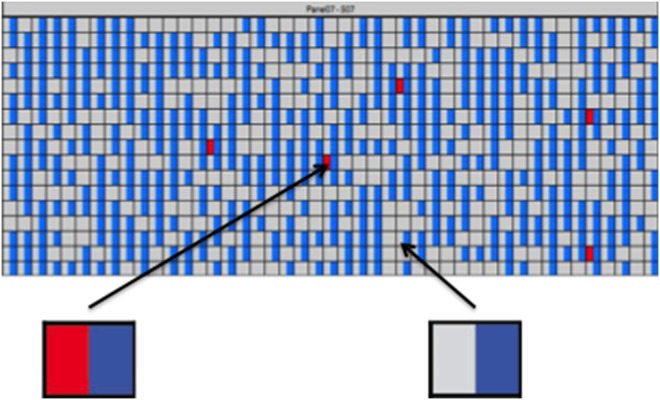
Determination of the detection limit of the D-PCR assay for T790M mutation. The assay panel included 765 chambers. A chamber harboring both red signal and blue signal represented T790M positive ones. Only blue signal in the chamber represented negative T790M mutation. This figure referred to the panel based status of T790M mutation when the mixed DNA (T790M mutant DNA fragments and internal control DNA fragments) with dilution concentration of 1∶3000.

Sensitivity assays were performed using the serially diluted T790M mutation in genomic DNA solutions (1∶100, 1∶300, 1∶1000, 1∶3000 and 1∶10000). ARMS were performed for DNAs isolated from individualized dots to confirm their positivity. The positive signal ratios (red/blue signals) for T790M in each DNA dilution were: 1∶100 (115/659), 1∶300 (20/686), 1∶1000 (15/692), 1∶3000 (5/702) (Figure 1), and 1∶10000 (0/686). Therefore, the detection limit of D-PCR for detecting the T790M mutation was approximately 0.03%.

### D-PCR was more sensitive than ARMS in qualitative T790M mutation

The T790M mutation was detected by ARMS and D-PCR, respectively, in pre-TKI and post-TKI plasma (Table 2). In pre-TKI plasma samples, ARMS detected T790M in only 5.5% of patients (6/103), whereas D-PCR detected T790M in 31.1% of patients (32/103). Of the 135 post-TKI plasma samples, D-PCR identified a higher frequency of T790M-positive samples than ARMS (43.0% vs. 25.2%). All T790M mutations detected by ARMS were also positive by D-PCR in our cohort.

**Table 2:** Comparison of T790M detected by ARMS and digital PCR in pre- and post-EGFR-TKI plasma samples.

	ARMS		D-PCR		
T790M	No.	%	No.	%	P
Pre-TKI (n = 103)	6	5.8	32	31.1	<0.001
Post-TKI (N = 135)	34	25.2	58	43.0	0.001

TKI: tyrosine kinase inhibitor.

Noticeably, among 32 patients identified with pre-TKI T790M mutation by D-PCR, 6 cases presented this gene aberration alone, accompanying with neither common sensitizing EGFR mutation (19del or 21L858R) nor uncommon mutation (e.g. G719x and S7681, et al) and the rest possessed concurrently EGFR mutations.

### The correlations of qualitative and quantitative T790M mutation in pre- and post-TKI plasma cf-DNA by D-PCR with survivals on EGFR-TKI

Among 103 pre-TKI plasma samples, 6 patients were T790M-positive by ARMS. The median PFS after EGFR-TKI among these patients was not significantly different from the median PFS of patients without T790M by ARMS (p = 0.308). However, the p-value reached statistical significance when D-PCR was used for T790M detection. T790M-positive patients showed an inferior median PFS (8.9 vs. 12.1 months, p = 0.007) and median OS (19.3 vs. 31.9 months, p = 0.001) compared with T790M-negative patients by D-PCR. In contrast, when post-TKI samples were analyzed, no correlations between T790M positivity and survival (PFS and OS) after EGFR-TKI were observed by either ARMS or D-PCR.

**Figure 2:**
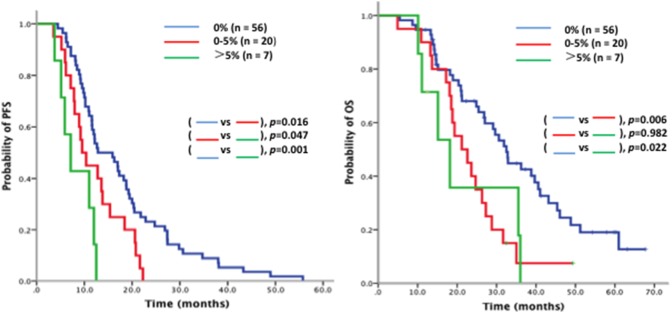
Survival analysis on EGFR-TKI according to the quantity of EGFR T790M mutation. Kaplan-Meier curves of (A) PFS and (B) OS according to the quantity of *de novo* EGFR T790M mutation identified by D-PCR in patients harboring sensitizing EGFR mutations (19del or 21L858R) (n = 83). The quantity of *de novo* T790M mutation was divided to three classes (high, low and nil), which referred to the quantity of *de novo* T790M mutation >5%, 0–5%, and 0%, respectively.

Given that sensitizing EGFR mutations (19del and L858R) are the most important predictor of the response to EGFR-TKI, de novo T790M mutation quantitative analysis was performed in patients with sensitizing EGFR mutations (n = 83). In the cohort of 83 cases, patients were subdivided into three groups based on the quantity of T790M in pre-TKI plasma samples by D-PCR (high: >5%, n = 7; low: 0–5%, n = 20; and nil: 0%, n = 56). The median PFS was 7.1 vs. 9.5 vs. 12.8 months (p = 0.001, Figure 2A). The median OS was 18.2 vs. 21.2 vs. 32.5 months (p = 0.005, Figure 2B). Subsequently, we analyzed the value of post-TKI T790M and failed to find correlations between qualification and quantitation of post-TKI T790M and survival (PFS and OS) with EGFR-TKI by D-PCR.

### Dynamic monitoring T790M quantity predicted the survival on EGFR-TKI

**Figure 3:**
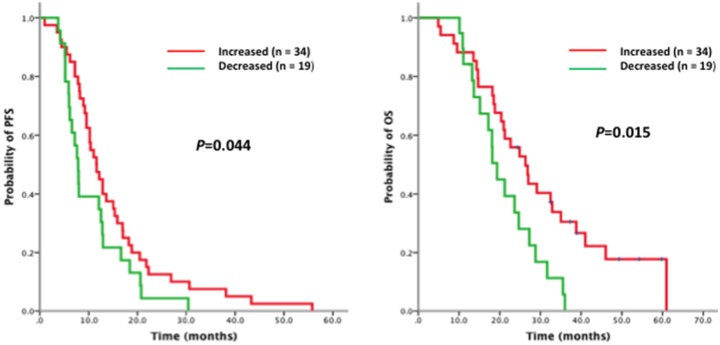
Survival analysis on EGFR-TKI according to the dynamic change of EGFR T790M mutation. Kaplan-Meier curves of (A) PFS and (B) OS according to the dynamic changing tendency of EGFR T790M quantity from pre- to post-EGFR-TKI treatment in patients with both T790M in pre- or post-EGFR-TKI plasma samples and sensitizing EGFR mutations (19del or 21L858R) in initial diagnosis tissue samples (n = 53).

A total of 103 patients had matching plasma samples collected before and after EGFR-TKI. Quantification of matched T790M samples indicated that 59 patients showed the same T790M status before and after EGFR-TKI (19 from positive to positive, and 40 from negative to negative). The other 44 patients showed different T790M status before and after EGFR-TKI (31 from negative to positive, and 13 from positive to negative) (Table 3). In the patient cohort with sensitizing EGFR mutations, patients with T790M either in pre-TKI or post-TKI samples (n = 53) were further subdivided into two groups (group 1, n = 34, increased quantity of T790M during EGFR-TKI; group 2, n = 19, decreased quantity of T790M during EGFR-TKI). Patients in group 1 showed superior PFS and OS compared with group 2 (PFS: 11.6 vs. 7.1 months, p = 0.044; OS: 26.3 vs. 19.3 months, p = 0.015, respectively) (Figures 3A and 3B).

**Table 3:** The variation tendency of T790M mutation from pre- to post-TKI in plasma cf-DNA.

	Pre-TKI T790M in cf-DNA		Case NO.
	*Pre-*positive	*Pre-*negative	
Post-TKI T790M in cf-DNA			
* Post-*positive	19	31	50
* Post-*negative	13	40	53
Case NO.	32	71	103

TKI: tyrosine kinase inhibitor.

Cf-DNA: cell-free DNA.

To identifying the effect of other resistant gene variation on the change of T790M quantity, we then detected c-MET amplification in post-EGFR-TKI plasma samples and found that 32% of patients harbored this gene variation in group 2 (6/19), and that only 12% of patients harbored a c-MET amplification in group 1 (4/34).

## Discussion

With the development of T790M-specific drugs such as AZD9291 [23], [24] and CO1686 [25], [26], issues have been raised regarding how to establish molecular detection platforms to accurately identify T790M-positive patients in whom a T790M inhibitor should be used to prevent resistance.

In the present study, we explored the availability of a noninvasive assay for T790M mutation in plasma cf-DNA, and reported the largest series on quantitative and dynamic monitoring T790M mutation in matched plasma samples before and after EGFR-TKI therapy. Our results indicated that D-PCR was a highly sensitive and useful method for detecting the T790M mutation. Qualitative and quantitative T790M in plasma cf-DNA can predict survival on EGFR-TKI. Moreover, dynamically monitoring T790M change might help determining EGFR-TKI prognosis.

As a highly sensitive and quantitative method, D-PCR had been used in the assessment of sensitizing EGFR mutations [17], [19]. In the present study, we used D-PCR to analyze the quality and quantity of the T790M mutation. Our findings indicated that the detection limit of D-PCR for qualitative T790M mutation was 0.03%. By D-PCR, we identified higher frequency of T790M compared with the conventional ARMS method in pre-TKI or post-TKI samples, which suggested that D-PCR was a highly sensitive method for identifying the T790M mutation.

An Increasing of studies including our previous’ have explored the availability of plasma cf-DNA for the assessment of gene variations such as sensitizing EGFR mutation [14]–[17] and KRAS mutation [27]. In the present study, we reported that the frequency of the T790M mutation was 31% and 43% in pre- and post-TKI plasma cf-DNA respectively, similarly with those observed in tumor tissue samples [10]–[12]. Recently, the concordance of T790M between tissue and plasma DNA after EGFR-TKI therapy had been reported to be 63% (17/27) [23] and 71% (5/7) [18] using the Cobas technology and DNA sequencing, respectively. We also analyzed the T790M mutation in 11 pairs of matched plasma cf-DNA and tumor tissues, and obtained 64% of concordance and 100% of specificity (data not shown).Together with previous and our results, we suggested the availability of plasma cf-DNA for EGFR T790M detection, which offered a promising strategy for T790M detection.

By utilizing D-PCR to analyze the T790M mutation status in plasma cf-DNA, we demonstrated that patients with de novo T790M showed inferior PFS compared with those without T790M, whether stratified by sensitizing EGFR mutations or not. Several studies by Maheswaran et al [12], Rosell et al [5] and Su et al [11] also indicated that de novo T790M was associated with poor outcomes of EGFR-TKI therapy. However, some studies showed emergence of T790M in acquired resistant tumor tissues was associated with superior prognosis after EGFR-TKI compared with those without T790M [29]–[31]. Cancer cells harboring T790M represent not only resistance to EGFR-TKI but also clones proliferating relatively slowly [32], which suggested that de novo T790M predicted poor prognosis after EGFR-TKI and acquired T790M conferred superior prognosis after a long period of enrichment of T790M mutant clones. So, our results were not contrary to previous observations. Besides high sensitivity, D-PCR provided an available strategy to accurately estimate the quantity of gene mutation [17], [19]. Using this approach, we further analyzed the quantity of T790M in pre-TKI plasma samples. In the subset of EGFR mutant patients, high quantity of T790M predicted inferior PFS after EGFR-TKI compared with low ones. These results suggested that the quantity of T790M may influence the clinical outcomes of EGFR-TKI treatment. Taken together, our findings highlighted the role of de novo T790M quantitative analysis in predicting PFS after EGFR-TKI, and might support early administration of T790M-specific inhibitors [23]–[26].

Noninvasive genotyping in plasma cf-DNA facilitates the dynamic monitoring of gene mutation during treatment, which was important in optimizing personalized therapy [28], [33]. Herein, we evaluated the correlation between survival with EGFR-TKI therapy and dynamic changes in the quantity of the T790M in matched plasma samples before and after EGFR-TKI therapy. Among EGFR-mutant cases, patients with an increasing trend in T790M quantity from pre- to post-EGFR-TKI displayed superior PFS and OS compared with patients with a decreasing trend. A likely explanation for these findings was that the resistance process was torpid during the enrichment of resistant cancer cells carrying *de novo* T790M mutation, which leaded to a long EGFR-TKI sensitive phase. To understand why patients with a decreasing trend in T790M quantity from pre- to post-TKI displayed inferior survival with EGFR-TKI treatment, we analyzed c-MET amplification in these patients and found that a higher rate of c-MET amplification in T790M-decreased patients than T790M-increased ones. These results suggested that cancer cells harboring other driver genes, such as c-MET, may rapidly proliferate, inducing resistance to EGFR-TKI, and diluting the concentration of the T790M mutation. However, these speculations need to be confirmed in basic researches.

Although the EGFR T790M mutation was considered to occur in patients carrying an sensitizing EGFR mutation [6], [7], recent studies showed that this molecular aberrance may also occur alone [34]. In the present study, 6 patients were identified to harbor T790M but not EGFR sensitive mutation or uncommon mutation, which suggested that T790M might be an independent molecular event excluded with other driver genes in some instances.

In summary, utilizing D-PCR in plasma cf-DNA provided a noninvasive and promising assay to qualitative and quantitative T790M mutation. By this approach, relatively high frequency of T790M could also be detected in plasma samples, thereby facilitating the dynamic detection of the T790M mutation. T790M in EGFR-TKI naïve samples was a negative predictive factor for survival on EGFR-TKI. Through quantitative assays, high quantity of *de novo* T790M in pre-TKI plasma samples predicted poor survival on EGFR-TKI. Moreover, dynamic monitoring the change of T790M quantity might help determining EGFR-TKI prognosis.
